# Assessment of cardiac safety during fingolimod treatment initiation in a real-world relapsing multiple sclerosis population: a phase 3b, open-label study

**DOI:** 10.1007/s00415-013-7115-8

**Published:** 2013-11-13

**Authors:** Ralf Gold, Giancarlo Comi, Jacqueline Palace, Arno Siever, Rebecca Gottschalk, Mahendra Bijarnia, Philipp von Rosenstiel, Davorka Tomic, Ludwig Kappos, For the FIRST Study Investigators

**Affiliations:** 1 Department of Neurology, St Josef Hospital, Ruhr-University, Gudrunstr. 56, 44791 Bochum, Germany; 2 Department of Neuroscience, San Raffaele Scientific Institute, University of Milan, Milan, Italy; 3 Department of Clinical Neurology, Oxford University Hospitals Trust, Oxford, UK; 4 Gemeinschaftspraxis, Oldenburg, Germany; 5 Novartis Pharmaceuticals Corporation, East Hanover, NJ USA; 6 Novartis Healthcare Pvt Ltd, Hyderabad, India; 7 Novartis Pharma AG, Basel, Switzerland; 8 Department of Neurology, University Hospital, Basel, Switzerland

**Keywords:** Fingolimod, Multiple sclerosis, Safety, Tolerability

## Abstract

The aim of this study was to evaluate short-term safety and tolerability of fingolimod in a real-world population with relapsing multiple sclerosis, focusing on cardiac safety during treatment initiation. Patients received fingolimod 0.5 mg once daily for four months. Patients excluded from the pivotal studies with certain pre-existing cardiac conditions or baseline cardiac findings (PCCs), and those receiving beta blockers (BBs) and/or calcium channel blockers (CCBs), were eligible. Heart rate (HR) and electrical conduction events were monitored using ambulatory electrocardiography for at least 6 h after the first dose. Of 2,417 enrolled patients, 2,282 (94.4 %) completed the study. Fingolimod initiation was associated with a transient, mostly asymptomatic decrease in HR. Bradycardia adverse events occurred in 0.6 % of patients and were more frequent in individuals receiving BBs/CCBs (3.3 %) than in other patient subgroups (0.5–1.4 %); most events were asymptomatic, and all patients recovered without pharmacological intervention. In the 6 h post-dose, the incidences of Mobitz type I second-degree atrioventricular block (AVB) and 2:1 AVB were higher in patients with PCCs (4.1 and 2.0 %, respectively) than in those without (0.9 and 0.3 %, respectively); at pre-dose screening, patients with PCCs had the same incidence of Mobitz type I second-degree AVB (4.1 %) and a slightly lower incidence of 2:1 AVB (0.7 %) than 6 h post-dose. All recorded conduction abnormalities were asymptomatic. This study adds to the evidence showing that cardiac effects during fingolimod initiation remain consistent with those known from previous, controlled studies, even if patients with PCCs are included.

**Electronic supplementary material:**

The online version of this article (doi:10.1007/s00415-013-7115-8) contains supplementary material, which is available to authorized users.

## Introduction

Fingolimod (FTY720) 0.5 mg once daily (Gilenya™, Novartis Pharma AG, Basel, Switzerland), a sphingosine 1-phosphate receptor (S1PR) modulator [1], was the first oral therapy approved for relapsing multiple sclerosis (MS) [2–5].

Fingolimod has shown superior efficacy to intramuscular interferon β-1a in a 1-year study and to placebo in two 2-year phase 3 studies [6–8]. These pivotal studies also established fingolimod as a treatment option with favorable safety and tolerability.

Administration of the first dose of fingolimod is associated with a decrease in heart rate (HR) and slowing of atrioventricular (AV) conduction. This is a recognized pharmacological effect of fingolimod, mediated by modulation of S1PR subtype 1 (S1P_1_) on atrial myocytes, which is similar to vagal stimulation. The effect is typically transient, owing to the internalization/desensitization of S1P_1_ [9], leading to functional antagonism rather than agonism. The pivotal studies were designed to specifically assess these effects in patients with relapsing MS and showed that the first-dose effects of fingolimod on HR and rhythm were transient (most resolved spontaneously within 24 h), and that the symptoms of conduction delays were uncommon (reported in <1 % of patients) and typically mild or moderate in severity [6–8]. However, as these studies were conducted when fingolimod was an experimental therapy, the patient criteria were cautious, excluding patients with pre-existing cardiac conditions and concomitant treatment with common HR-lowering drugs.

Accordingly, this phase 3b, open-label, Fingolimod Initiation and caRdiac Safety Trial (FIRST) evaluated the short-term safety and tolerability of fingolimod 0.5 mg, focusing in particular on cardiac safety during treatment initiation, in a broader population of patients with relapsing MS than previously investigated in the clinical development program [6–8, 10]. It did not exclude patients with a history of cardiac conditions (such as recurrent bradycardia) or those receiving HR-lowering drugs [such as beta blockers (BBs) or calcium channel blockers (CCBs)] to establish whether the results of the pivotal studies remain consistent in a population that more closely reflects the patients who present in everyday clinical practice.

## Methods

### Study oversight

FIRST (ClinicalTrials.gov Identifier: NCT01497262) was conducted in accordance with the International Conference on Harmonisation Guidelines for Good Clinical Practice [11] and the Declaration of Helsinki [12]. The protocol was approved by each site’s independent Ethics Committee or Institutional Review Board. All patients gave written informed consent before any study-related procedures were performed.

### Participants

Patients were eligible for participation if they had a diagnosis of relapsing MS according to the 2005 revised McDonald criteria [13], were aged 18–65 years, and had an Expanded Disability Status Scale score of 0–6.5 [14]. In contrast to the clinical development studies, participants with controlled diabetes mellitus and the following pre-existing cardiac conditions or baseline cardiac findings (PCCs) were also eligible: recurrent symptomatic bradycardia, resting pulse rate of 45–54 beats per minute (bpm), history of a positive tilt test for vasovagal syncope, and history or presence of Mobitz type I second-degree AV block (AVB) on the screening or baseline electrocardiogram (ECG). Concomitant medication with BBs and other HR-lowering drugs was permitted. Patients were excluded if they had previously participated in a fingolimod study or had any cardiac disorder other than those stated above. Other exclusion criteria were consistent with previous fingolimod trials and have been described elsewhere [6–8, 10].

### Study design

FIRST was a 4-month, open-label, single-arm, phase 3b, multicenter study that was conducted between May 2010 and October 2011 at 285 study centers across 23 countries. All patients were treated with fingolimod 0.5 mg for 16 weeks. Patients initiated treatment either on-site (clinic or office setting) or off-site.

### Study procedures and endpoints

#### Screening

Patients entered a 1 to 2-week screening phase to determine eligibility; screening included a 24-h ambulatory ECG (AECG) recording and an ECG, both of which were analyzed by the central ECG vendor. The following safety assessments were carried out: physical examination, vital sign recordings, laboratory evaluations, pregnancy testing for women, and ophthalmological examination and optical coherence tomography performed by an ophthalmologist.

#### Before fingolimod first-dose administration

A pre-dose ECG was performed to establish whether patients had PCCs; if they did, it was recommended that those patients were monitored on-site rather than off-site. However, any patient (with or without PCCs) could be monitored on-site if the local investigator considered this appropriate.

#### Fingolimod treatment initiation

It was recommended that all patients take their first dose of fingolimod before noon. All patients’ HRs and electrical conduction events were monitored via AECG recording for at least 6 h post-dose. Additionally, for individuals monitored on-site, vital signs were recorded hourly for approximately 6 h post-dose, and patients were asked to inform the investigator about any cardiac symptoms or other adverse events (AEs) that they experienced. Patients initiating therapy off-site were instructed to contact the investigator if any AEs occurred.

Patients monitored on-site were discharged after 6 h providing that their HR was >80 % of the baseline value and was not the lowest observed value, and that they had no symptoms associated with decreased HR. If these discharge criteria were not met, patients were monitored hourly until the criteria were fulfilled. If an individual had an HR <40 bpm, they were observed overnight on a ward/monitoring unit. If a patient’s HR decreased more than 30 % from baseline at any time during the first 6-h monitoring period, or if they had any symptomatic event associated with a reduction in HR during the first 24 h, the second dose of fingolimod 0.5 mg was to be taken on-site under first-dose monitoring conditions.

#### Open-label treatment phase

Safety assessments were performed at weeks 2, 8 and 16. The AEs, serious AEs (SAEs), concomitant medication, vital signs, hematology, blood chemistry, pregnancy test results for women, and MS relapses were assessed at each study time point. Ophthalmological examinations were performed at 16 weeks. Patients who completed the 16-week treatment phase could enter an optional extension study to receive continuous treatment with fingolimod 0.5 mg. Patients who did not enter the extension returned for a follow-up visit 12 weeks after the last dose of study drug for final safety assessments.

#### Endpoints

The primary objective of FIRST was to evaluate the short-term safety and tolerability profile of fingolimod 0.5 mg in a broader population of patients with relapsing forms of MS than previously studied with a focus on cardiac safety. The variables used to evaluate the primary objective were: AEs and SAEs; laboratory, vital sign and first-dose AECG recording data; and ophthalmological and skin assessments.

### Statistical analyses

The incidence of AVBs and conduction abnormalities recorded by the first-dose 6-h ECG, alanine aminotransferase elevations determined by liver function tests, and macular edema diagnosis were selected for the consideration of sample size calculation (see online resource 1). Primary variables were summarized using descriptive statistics. For continuous variables, summary statistics included number, mean, standard deviation, and minimum and maximum values; frequencies and percentages were provided for categorical variables. The AECG data were analyzed to determine the change in mean HR from the equivalent hour at screening to 6 h post-dose. Vital signs and laboratory data were summarized as mean change from baseline. Supportive safety analyses were summarized for the following subgroups: patients with PCCs (PCC group) versus those who did not have pre-existing cardiac conditions or baseline cardiac findings [non-PCC (NPCC) group], patients co-medicated with BBs and/or CCBs (BB/CCB group) versus those who were not (NBB/NCCB group), and patients with diabetes mellitus versus those without.

## Results

### Study population

Of 2,417 enrolled patients, 2,415 received at least one dose of the study drug and were included in the safety set. A total of 2,282 patients (94.4 %) completed, and 135 individuals (5.6 %) discontinued, the study, most commonly owing to AEs (2.8 %), abnormal laboratory values (1.1 %), or withdrawal of consent (0.5 %).

Baseline demographic and clinical characteristics (Table 1) were consistent with a typical population of patients with relapsing MS. Most participants (85 %) had been previously treated with disease-modifying therapy (Table 1). Approximately 50 % of patients were monitored off-site.

**Table 1:** Patient demographic and baseline clinical characteristics, previous treatment with DMTs and type of first-dose monitoring in the overall population and subgroups (enrolled population)

	Fingolimod 0.5 mg (*n* = 2,417)
Female, *n* (%)	1,773 (73.4)
Age groups, years, *n* (%)	
18–30	552 (22.8)
31–40	804 (33.3)
41–55	981 (40.6)
56–65	80 (3.3)
Duration of MS since first symptom, years, mean ± SD	9.3 ± 6.9
Number of relapses in past year, mean ± SD	1.1 ± 1.1
EDSS score, mean ± SD	2.4 ± 1.5
History of macular edema, *n* (%)	2 (0.1)
History of uveitis, *n* (%)	25 (1.0)
History of optic neuritis, *n* (%)	1,022 (42.3)
Patients with diabetes mellitus, *n* (%)	26 (1.1)
Previously treated with DMTs, *n* (%)	2,054 (85)
Any interferon β	1,699 (70.3)
Other interferons	636 (26.3)
Glatiramer acetate	747 (30.9)
Natalizumab	254 (10.5)
Azathioprine	86 (3.6)
Methotrexate	6 (0.2)
Other MS medications	123 (5.1)
Patients monitored on-site, *n* (%)^a^	1,221 (50.5)
Patients with PCCs, *n* (%)	296 (12.2)
Patients with PCCs who were monitored on-site, *n* (%)	271 (11.2)
Patients receiving concomitant treatment with BBs/CCBs, *n* (%)	120 (5.0)
Patients receiving concomitant treatment with BBs/CCBs who were monitored on-site, *n* (%)	78 (3.2)

*BBs* beta blockers, *CCBs* calcium channel blockers, *DMT* disease-modifying therapy, *EDSS* Expanded Disability Status Scale, *MS* multiple sclerosis, *PCCs* pre-existing cardiac conditions or baseline cardiac findings, *SD* standard deviation

^a^Two of these patients did not receive study drug and were, therefore, not included in the safety set

### Cardiac effects during treatment initiation

#### Adverse events and serious adverse events

Cardiac AEs were reported in 49/2,415 patients (2.0 %) in the overall study population on days 1 and 2 after treatment initiation; 14 of these patients were in the PCC or BB/CCB subgroups (Table 2). Bradycardia was the most frequently reported cardiac AE (15 patients, 0.6 %), with proportionately more cases occurring in the BB/CCB group (3.3 %) than in the other subgroups (0.5–1.4 %) (Table 2); these cases were mostly asymptomatic and all patients recovered without pharmacological intervention. Four patients (0.2 %) discontinued fingolimod owing to a cardiac AE [suspected angina pectoris (day 1 and day 33), Mobitz type I second-degree AVB (day 1), and cardiovascular disorder (day 3)]; none of these patients had PCCs or were co-medicated with BBs/CCBs.

**Table 2:** Cardiac adverse events occurring in ≥1 patient on days 1 and 2 after administration of first dose of fingolimod 0.5 mg by subgroups (safety set)

Number of patients (%)	Overall (*n* = 2,415)	No PCCs (*n* = 2,120)	PCCs (*n* = 295)	No BBs/CCBs (*n* = 2,295)	BBs/CCBs (*n* = 120)
Total	49 (2.0)	42 (2.0)	7 (2.4)	42 (1.8)	7 (5.8)
Palpitations	14 (0.6)	14 (0.7)	0 (0.0)	14 (0.6)	0 (0.0)
Bradycardia	15 (0.6)	11 (0.5)	4 (1.4)	11 (0.5)	4 (3.3)
Tachycardia	2 (0.1)	1 (0.0)	1 (0.3)	2 (0.1)	0 (0.0)
Cardiovascular disorder	3 (0.1)	3 (0.1)	0 (0.0)	3 (0.1)	0 (0.0)
Angina pectoris	3 (0.1)	2 (0.1)	1 (0.3)	2 (0.1)	1 (0.8)
Second-degree AVB	5 (0.2)	5 (0.2)	0 (0.0)	5 (0.2)	0 (0.0)
Ventricular extra systoles	4 (0.2)	3 (0.1)	1 (0.3)	2 (0.1)	2 (1.7)
Ventricular tachycardia	1 (0.0)	1 (0.0)	0 (0.0)	1 (0.0)	0 (0.0)
Cardiac disorder	1 (0.0)	1 (0.0)	0 (0.0)	1 (0.0)	0 (0.0)
Sinus bradycardia	1 (0.0)	1 (0.0)	0 (0.0)	1 (0.0)	0 (0.0)
AVB	1 (0.0)	1 (0.0)	0 (0.0)	1 (0.0)	0 (0.0)

Safety set includes all patients who received at least one dose of fingolimod

Extra systoles, arrhythmia, first-degree AVB, left or right bundle branch block, cardiac asthma or left ventricular hypertrophy did not occur in any patients

*AVB* atrioventricular block, *BBs* beta blockers, *CCBs* calcium channel blockers, *PCCs* pre-existing cardiac conditions or baseline cardiac findings

During the 4-month study, six SAEs reported in five patients were classified as cardiac disorders: angina pectoris, second-degree AVB, bradycardia, cardiac disorder, cardiovascular disorder, and sinus bradycardia. None of the patients with a cardiac SAE were in the PCC or BB/CCB subgroups. All events manifested within 48 h of receiving the first dose except for one that occurred 41 days post-dose (reported as cardiovascular disorder with moderate symptoms of dizziness, hypotension and nausea). Two of the cardiac SAEs (suspected, but not confirmed angina pectoris and Mobitz type I second-degree AVB, which was asymptomatic) resulted in study drug discontinuation. The suspected case of angina pectoris was reported in a 30-year-old woman: the event was reported on treatment initiation as mild in nature, occurred a few minutes after treatment initiation, but reoccurred with subsequent dosing; the event completely resolved upon treatment discontinuation and without intervention. The case of second-degree AVB was detected by Holter monitoring in a 24-year-old woman following treatment initiation: the event was asymptomatic, lasted for <3 s and was recorded as severe; treatment was discontinued. The cases of bradycardia and cardiac disorder detected on ECG were reported in a 23-year-old woman on day 2 who was diagnosed with a urinary tract infection and a white blood cell count increase the day before: the events were mild (cardiac disorder; manifested as chest pain and difficulty in breathing and micturition) or moderate (bradycardia) in severity and resolved without intervention on the same day and in the same timeframe as the infection while continuing fingolimod therapy. The case of cardiovascular disorder reported in a 40-year-old man on day 41 was manifest as circulation problems (considered probably to be a migraine), dizziness, tinnitus, vertigo and vomiting, which were all moderate in severity: hetastarch and metoclopramide were used to treat the symptoms and the patient continued treatment with fingolimod. The case of sinus bradycardia was reported on day 1 after treatment initiation in a 26-year-old man who had abnormal screening ECGs at day −17 (first-degree AVB and ventricular premature beats) and baseline (intraventricular conduction defect, first-degree AVB and sinus bradycardia): the event was moderate in severity, had resolved by the next day, did not require intervention, and the patient continued fingolimod therapy.

#### Incidence of AV conduction abnormalities recorded by AECG

For the overall population, the incidences of Mobitz type I second-degree AVB and 2:1 AVB in the 6 h post-dose were 1.3 and 0.5 %, respectively, compared with 0.5 and 0.1 %, in the 6 h pre-treatment. No patient developed Mobitz type II second-degree AVB or complete AVB. Mobitz type I second-degree AVB occurred approximately five times more frequently in patients with PCCs than in those without PCCs, both pre- and post-treatment initiation (Table 3). Although rare overall, new-onset AVB post-dose was detected twice as frequently in patients in the PCC group (2 %) than in the NPCC group (0.9 %). No AVBs were detected in the BB/CCB group, pre- or post-treatment initiation.

**Table 3:** Incidence of AVBs on AECG recording: 6 h pre-treatment versus following administration of first dose of fingolimod 0.5 mg by subgroups and by type of AVB (safety set)

Number of patients with events^a^ (%)	No PCCs (*n* = 2,120)	PCCs (*n* = 295)	BBs/CCBs (*n* = 120)
Pre-treatment AECG			
Mobitz type I second-degree AVB	0	12 (4.1)	0
2:1 AV block	0	2 (0.7)	0
Post-dose AECG			
Mobitz type I second-degree AVB	18 (0.9)	12 (4.1)	0
2:1 AVB	7 (0.3)	6 (2.0)	0
Patients with events both pre-dose and post-dose	0	6 (2.0)	0
Patients with new post-dose events	19 (0.9)	6 (2.0)	0

Safety set includes all patients who received at least one dose of fingolimod

*AECG* ambulatory electrocardiogram, *AVB* atrioventricular block, *BBs* beta blockers, *CCBs* calcium channel blockers, *PCCs* pre-existing cardiac conditions or baseline cardiac findings

^a^ Some individuals had two types of second-degree AVB (Mobitz type I and 2:1 AVB)

#### Pulse rate

**Fig. 1:**
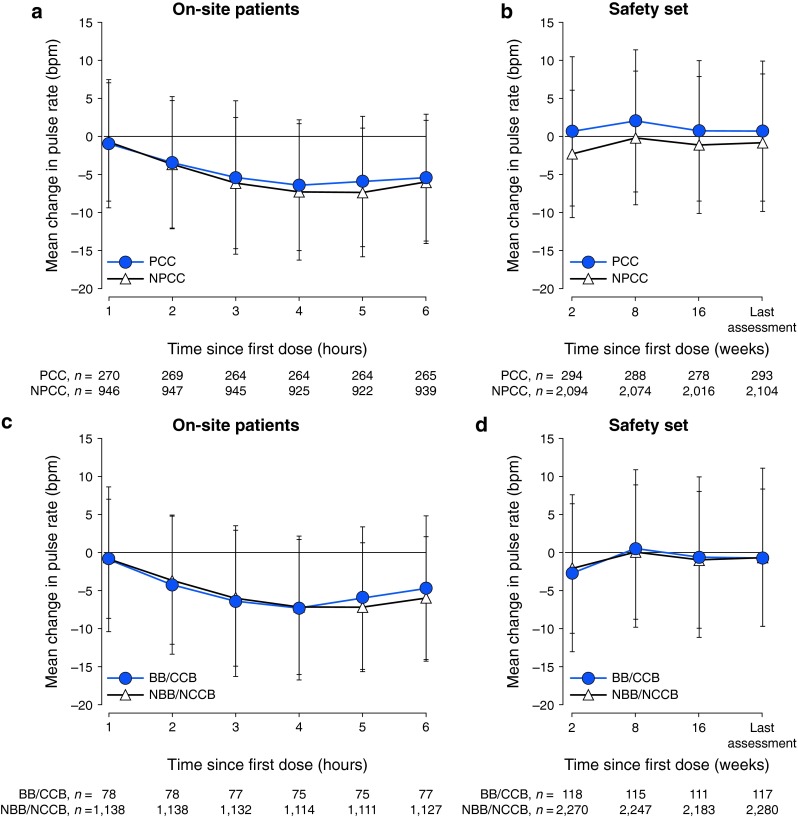
Hourly mean change in pulse rate from pre-dose sitting pulse rate (**a**) and mean change in pulse rate across visits from pre-dose sitting pulse rate (**b**) for patients who had/did not have pre-existing cardiac conditions or baseline cardiac findings (PCCs), and hourly mean change in pulse rate from pre-dose sitting pulse rate (**c**) and mean change in pulse rate across visits from pre-dose sitting pulse rate (**d**) for patients who were receiving/not receiving beta blockers/calcium channel blockers (BBs/CCBs). *bpm* beats per minute, *NBB/NCCB* not receiving BBs/CCBs, *NPCC* no PCCs

In the on-site population of 1,219 patients, the mean baseline pulse rate of the PCC group (68.6 bpm) was lower than that of the NPCC group (73.5 bpm). Patients in the PCC group reached a mean nadir pulse rate at 4 h post-dose compared with 5 h post-dose for patients in the NPCC group (Fig. 1). The maximum mean change in pulse rate was also smaller in the PCC group than in the NPCC group [−6.5 (range −36.3 to 17.3) bpm and −7.4 (range −45.7 to 23.3) bpm, respectively]. Mean baseline pulse rate was lower in the BB/CCB group (69.4 bpm) than in the NBB/NCCB group (73.0 bpm). The BB/CCB group reached a mean nadir pulse rate at 4 h post-dose compared with 5 h post-dose for the NBB/NCCB group. The maximum change in pulse rate from baseline was similar between the BB/CCB and NBB/NCCB groups [−7.3 (range −36.3 to 10) bpm and −7.2 (range −45.7 to 23.3) bpm, respectively]. Mean pulse rates for the on-site monitored patients are provided in online resource 2. Mean pulse rates over the 16-week treatment period were similar to pre-dose readings (variation of <5 bpm above or below pre-dose rates); a similar pattern was observed across subgroups.

Of the 1,219 on-site patients, 16 patients (1.3 %) had a reduction in HR to <45 bpm during the 6-h post-dose monitoring period. After 6 h, 40 patients (3.3 %) required extended monitoring. Of the 271 patients with PCCs who were monitored on-site, 15 patients (5.5 %) required extended monitoring. Of the 78 patients receiving BBs/CCBs who were monitored on-site, three patients (3.8 %) required extended monitoring. Most (31/40) patients who required extended monitoring were discharged after 7 h (i.e., after one additional hour of monitoring); this included 12 of the 15 patients with PCCs and all three patients receiving BBs/CCBs.

None of the nine patients who required extended monitoring beyond 7 h on day 1 discontinued fingolimod treatment due to a first dose event or were kept in for overnight monitoring. All nine patients were discharged on day 1 after a maximum of 3 h of extended monitoring. In eight of these patients, monitoring was extended on day 1 because the HR discharge criteria were not met (i.e., HR at 6 h post-dose was <80 % of the baseline pre-dose value or was the lowest observed value). Symptomatic bradycardia was reported in the remaining patient; this patient also did not meet the HR discharge criteria and returned for monitoring on day 2 before being discharged without extended monitoring or intervention. In one of the eight patients who required extended monitoring on day 1 because they did not meet the HR discharge criteria, an asymptomatic sinus bradycardia SAE was also reported on day 1 (details provided in the AE and SAE results section of this paper); the patient was hospitalized for second-dose monitoring and was discharged on day 2 without requiring intervention.

#### Heart rate on AECG at screening and after the first dose (safety set)

**Fig. 2:**
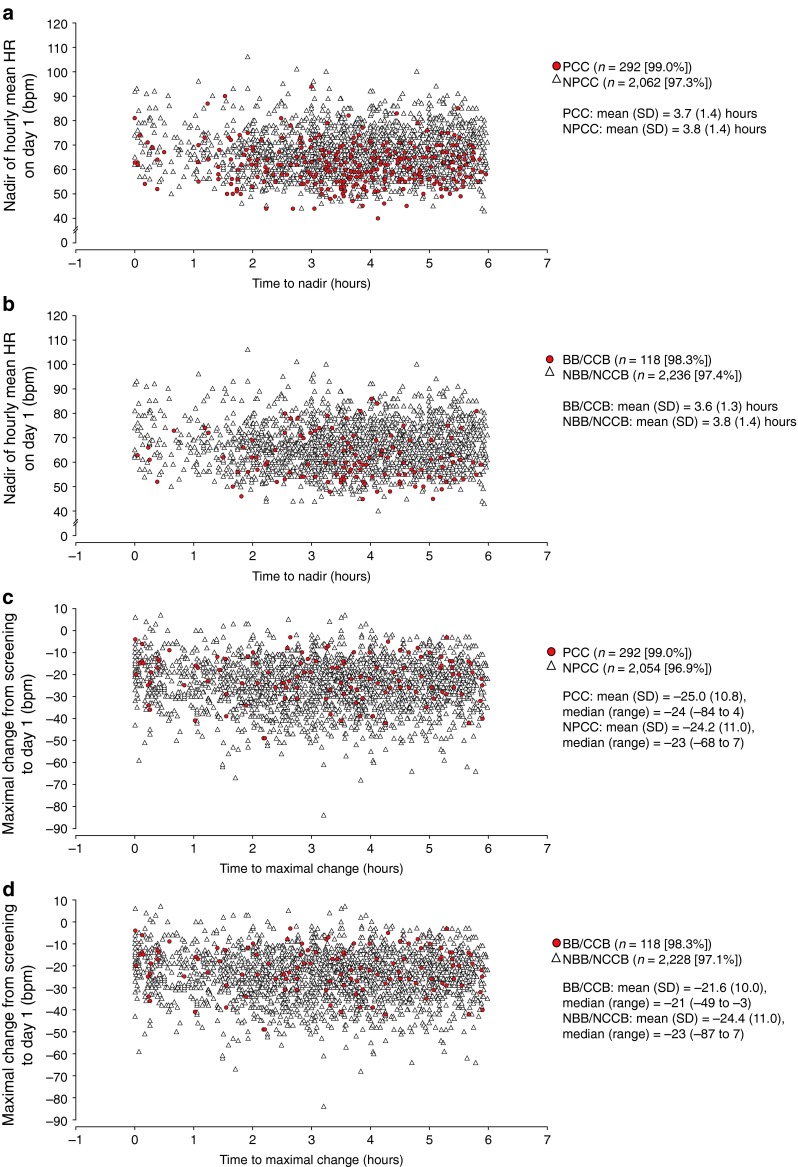
Time to nadir hourly heart rate (HR) in patients who had/did not have pre-existing cardiac conditions or baseline cardiac findings (PCCs) (**a**) and who were receiving/not receiving concomitant beta blockers/calcium channel blockers (BBs/CCBs) (**b**), and time to maximal change in HR 6 h following first-dose administration from equivalent time of day at screening for patients who had/did not have PCCs (**c**) and who were receiving/not receiving BBs/CCBs (**d**) Percentages were calculated using the total number of patients in each subpopulation (PCCs, *n* = 295; NPCCs, *n* = 2,120; BBs/CCBs, *n* = 120; NBB/NCCB, *n* = 2,295). *NBB/NCCB* not receiving BBs/CCBs, *NPCC* no PCCs, *SD* standard deviation

The mean time to nadir hourly HR was similar in patients with PCCs and those without (Fig. 2a). Similarly, the mean time to nadir hourly HR was comparable between patients receiving and not receiving BBs/CCBs (Fig. 2b). The maximum, mean and median changes in HR were slightly greater in the PCC group than in the NPCC group (Fig. 2c). The maximum, mean and median changes in HR in the BB/CCB group were smaller than in the NBB/NCCB group (Fig. 2d).

### Overall safety and tolerability

AEs and SAEs were reported in 75.3 % and 4.1 % of patients, respectively; details are provided in online resource 2. One death (suicide) occurred during the study and was not considered by the investigator to be related to the study drug. The most common AEs (reported in at least 5 % of patients) were nasopharyngitis, headache, lymphopenia, and fatigue. Results from dilated ophthalmoscopy assessments, laboratory evaluations, and details of other events of interest are provided in online resource 3. Mean systolic/diastolic blood pressure increased from 120.1/77.3 mmHg at baseline to 122.3/78.8 mmHg at month 2 and remained stable thereafter; over the 4-month study period, the mean increase was 2.2 (standard deviation [SD], 10.8)/1.3 (SD, 8.1) mmHg.

## Discussion

Consistent with previous studies [7, 8, 10, 15], the first dose of fingolimod was associated with a transient, mostly asymptomatic, decrease in HR. A larger maximal change in HR was observed with AECG monitoring in the whole population than with pulse rate data from the patients monitored on-site. The maximal change in pulse rate was measured as a change from baseline immediately post-dose in on-site patients who were sedentary. In contrast, HR changes on AECG recordings were based on post-dose hourly mean HR changes from the equivalent time of day at screening. Different levels of physical activity during screening, and after the first dose of fingolimod, might account for the larger change in HR on AECG recordings.

Although the occurrence of bradycardia was rare in the overall study population, more cases of bradycardia were observed in the BB/CCB group during the first two days of treatment than in other subgroups. The lower baseline mean pulse rate observed in the BB/CCB group than in the NBB/NCCB group (as expected with these medications) may have potentially predisposed these patients to bradycardia. It has been shown previously in healthy volunteers that atenolol in combination with fingolimod results in a 15 % lower nadir HR than fingolimod alone [16]. Interestingly, despite more cases of bradycardia in the BB/CCB subgroup, the mean reduction in pulse rate and timing of recovery was generally similar between patients in the BB/CCB and NBB/NCCB groups. The majority of patients did not require extended monitoring and were discharged after 6 h.

The overall incidence of AVBs following treatment initiation was low. A small number of AVBs occurred pre-treatment; emphasizing that AVBs can occur even without treatment and may go unnoticed. Mobitz type I second-degree AVBs and 2:1 AVBs occurred more frequently in the PCC group than in the NPCC group, both pre-dose and in the first 6 h post-dose. New-onset AVBs post-dose were more common in the PCC than in the NPCC group, although they were infrequent overall. Consistent with previous findings, conduction abnormalities were asymptomatic and no patients developed a Mobitz type II second-degree AVB or complete AVB. No patients in the BB/CCB group had any AVB detected during the 6-h pre- and post-dose AECG monitoring; however, this subgroup was small. No patients in the PCC or BB/CBB groups discontinued the study drug owing to a cardiac AE.

This is the first fingolimod clinical study that has extended eligibility to patients with PCCs. Overall, no new safety findings were identified using fingolimod 0.5 mg in this broader population of patients with MS compared with previous fingolimod studies with more restrictive eligibility criteria. The study findings confirm that cardiac effects following the first dose of fingolimod are transient, mostly asymptomatic, and observed in the first 6 h post-dose; this is consistent with previous studies [7, 8, 15]. Our findings further suggest that there is no increased risk of symptomatic or serious cardiac events during treatment initiation in patients with PCCs or in those receiving BBs/CCBs. A recent study (of 317 patients) in clinical practice also found that individuals using BBs or CCBs had uneventful first-dose observations [17]. This notwithstanding, fingolimod is not recommended for patients with a history of certain cardiac conditions that were included in FIRST or for patients who are receiving concurrent treatment with BBs/CCBs [2, 18].

Overall, the types of AEs and SAEs, as well as the low discontinuation rates observed in FIRST, were consistent with those seen in previous phase 3 fingolimod studies [6–8]. Specifically, the AEs of interest (macular edema, herpes infections, and liver enzyme elevations) did not show an increase over background rates observed in previous studies.

Although FIRST included a broader population of patients with MS than previously investigated and had a large sample size, it had some limitations. FIRST had no control group and the duration of the study was relatively short compared with other phase 3 studies. However, the aim of this study was to investigate early dosing with fingolimod, with a specific focus on HR and rhythm disturbances during treatment initiation. Owing to small and uneven group sizes, inferential statistical testing was not performed. Lastly, it should be acknowledged that only 26 patients with diabetes mellitus were included; therefore, definite conclusions should not be drawn regarding the risk of macular edema in this subpopulation.

FIRST extends our knowledge of the previously reported short-term safety and tolerability profile of fingolimod, in relation to both first-dose effects and the first four months of treatment, and includes a broader population of patients with relapsing MS than previously evaluated in the fingolimod development program. The results support the use of fingolimod in patients with relapsing MS who are more representative of those treated in everyday clinical practice than the phase 3 populations.

## Electronic supplementary material

Below is the link to the electronic supplementary material.

Supplementary material 1 (DOCX 30 kb)
