# Supplementary material for: Assessment of cardiac safety during fingolimod treatment initiation in a real-world relapsing multiple sclerosis population: a phase 3b, open-label study
Source: J Neurol. 2013 Nov 13;261(2):267–76. doi: 10.1007/s00415-013-7115-8 (PMC3915082; doi:10.1007/s00415-013-7115-8)
Supplement: Supplementary file 1 — Supplementary material 1 (DOCX 30 kb) [file 415_2013_7115_MOESM1_ESM.docx]

**Assessment of cardiac safety during fingolimod treatment initiation in a real-world relapsing multiple sclerosis population: a phase 3b, open-label study**

Ralf Gold,^1^ Giancarlo Comi,^2^ Jacqueline Palace,^3^ Arno Siever,^4^ Rebecca Gottschalk,^5^ Mahendra Bijarnia,^6^ Philipp von Rosenstiel,^7^ Davorka Tomic,^7^ Ludwig Kappos,^8^ for the FIRST Study Investigators

^1^Department of Neurology, St Josef Hospital, Ruhr-University, Bochum, Germany; ^2^University of Milan, Department of Neuroscience, San Raffaele Scientific Institute, Milan, Italy; ^3^Department of Clinical Neurology, Oxford University Hospitals Trust, Oxford, UK; ^4^Gemeinschaftspraxis, Oldenburg, Germany; ^5^Novartis Pharmaceuticals Corporation, East Hanover, NJ, USA; ^6^Novartis Healthcare Pvt Ltd, Hyderabad, India; ^7^Novartis Pharma AG, Basel, Switzerland; ^8^Department of Neurology, University Hospital, Basel, Switzerland

**Corresponding author:**

Ralf Gold, Department of Neurology, St Josef Hospital, Ruhr-University, Gudrunstr. 56, 44791 Bochum, Germany

Telephone: ++49 - (0)234 - 509 - 2410

Fax: ++49 - (0)234 - 509 - 2414

e-mail: [ralf.gold@ruhr-uni-bochum.de](http://www.rd.ruhr-uni-bochum.de/neuro/wiss/sprecher/ralf.gold@ruhr-uni-bochum.de)

**Online Resource 1**

Based on previous phase 3 fingolimod studies, a sample size of 2,400 patients allows the estimation of an overall rate of AVBs of 3% in the total study population with a 0.68% 1-sided confidence interval (CI) and an estimate of incidence of any conduction abnormality of 9% (95% CI: 7.9–10.1%) in the total population. For alanine aminotransferase elevations, a sample size of 2,160 patients provides an estimate of incidence of 5.8% (95% CI: 4.8–6.8%). Using pooled 6-month data from 854 patients receiving fingolimod 0.5 mg, an incidence of macular edema of 1.3% was estimated based on the assumption that approximately 10% of the enrolled population had diabetes mellitus and therefore had an associated higher background risk of developing macular edema. However, in this study, the proportion of patients with a diagnosis of diabetes mellitus was below 10%. Therefore, despite the revised sample size of 2,160 completed patients, an accurate estimation of the risk of macular edema was not possible in this study based on the incidence of macular edema in patients receiving the 0.5 mg dose of fingolimod during the phase 3 program in MS.

**Online Resource 2**

**Table 1** Adverse events (AEs) leading to study drug discontinuation in ≥0.2% of patients, AEs in >5% of patients by preferred term or primary system organ class, and serious AEs (SAEs) by primary system organ class in ≥0.2% of patients (safety set)

| **Number of patients (%)** | **Fingolimod 0.5 mg**  **(*n* = 2,415)** |
| --- | --- |
| Any AE | 1,819 (75.3) |
| Deaths | 1 (0.0) |
| Study drug discontinuations due to  Any AEs | 98 (4.1) |
| Any AEs in ≥0.2% of patients by preferred term |  |
| Macular edema | 13 (0.5) |
| Elevated liver enzymes^a^ | 10 (0.4) |
| Lymphopenia^b^ | 9 (0.4) |
| Liver function test abnormal^c^ | 4 (0.2) |
| Cardiac AE | 4 (0.2) |
| Drug-related AEs | 84 (3.5) |
| SAEs | 26 (1.1) |
| Abnormal laboratory values | 27 (1.1) |
| AEs occurring in ≥5% of patients by preferred term |  |
| Nasopharyngitis | 358 (14.8) |
| Headache | 273 (11.3) |
| Lymphopenia | 228 (9.4) |
| Fatigue | 160 (6.6) |
| AEs occurring in >5% of patients by primary  system organ class |  |
| Infections and infestations | 828 (34.3) |
| Nervous system disorders | 561 (23.2) |
| Gastrointestinal disorders | 396 (16.4) |
| General disorders and administration site conditions | 311 (12.9) |
| Investigations | 277 (11.5) |
| Musculoskeletal and connective tissue disorders | 272 (11.3) |
| Blood and lymphatic system disorders | 264 (10.9) |
| Skin and subcutaneous tissue disorders | 259 (10.7) |
| Psychiatric disorders | 177 (7.3) |
| Respiratory, thoracic, and mediastinal disorders | 160 (6.6) |
| Eye disorders | 140 (5.8) |
| Any SAE | 99 (4.1) |
| SAEs occurring in ≥0.2% of patients by primary system organ class |  |
| Nervous system disorders | 24 (1.0) |
| Multiple sclerosis relapse | 6 (0.2) |
| Infections and infestations | 19 (0.8) |
| Psychiatric disorders | 10 (0.4) |
| Depression | 4 (0.2) |
| Injury, poisoning, and procedural complications | 9 (0.4) |
| Eye disorders | 8 (0.3) |
| Macular edema | 5 (0.2) |
| Ear and labyrinth disorders | 6 (0.2) |
| Vertigo | 4 (0.2) |
| Gastrointestinal disorders | 6 (0.2) |
| Neoplasms benign, malignant, and unspecified^d^ | 6 (0.2) |
| Cardiac disorders | 5 (0.2) |
| Musculoskeletal connective tissue disorders | 5 (0.2) |
| Vascular disorders | 5 (0.2) |

^a^Increase in alanine aminotransferase (ALT) or aspartate aminotransferase (AST) > 5 × upper limit of the normal range (ULN)

^b^Any occurrence of lymphocyte count < 0.2 × 10^9^/L

^c^ALT > 90 U/L^;^ AST > 82 U/L; total bilirubin 2.0 mg/dL

^d^A total of 10 malignancies was reported; 4 were reported as AEs and 6 as SAEs

**Table 2** Mean pulse rates during the 6 hours after first-dose administration for the on-site population subgroups

| **Number of patients (%)** | **On-site**  **(*n* = 1,219)** | **No PCCs**  **(*n* = 948)** | **PCCs**  **(*n* = 271)** | **No BBs/CCBs**  **(*n* = 1,141)** | **BBs/CCBs**  **(*n* = 78)** |
| --- | --- | --- | --- | --- | --- |
| Lowest pulse rate (bpm) |  |  |  |  |  |
| <45 | 16 (1.3) | 4 (0.4) | 12 (4.4) | 14 (1.2) | 2 (2.6) |
| ≥45–54 | 179 (14.7) | 112 (11.8) | 67 (24.7) | 164 (14.4) | 15 (19.2) |
| ≥55–64 | 605 (49.6) | 475 (50.1) | 130 (48.0) | 566 (49.6) | 39 (50.0) |
| ≥65 | 419 (34.4) | 357 (37.7) | 62 (22.9) | 397 (34.8) | 22 (28.2) |
| Absolute change from pre-dose mean value (bpm) |  |  |  |  |  |
| < –40 | 4 (0.3) | 2 (0.2) | 2 (0.7) | 3 (0.3) | 1 (1.3) |
| ≥ –40 to –31 | 13 (1.1) | 10 (1.1) | 3 (1.1) | 12 (1.1) | 1 (1.3) |
| > –31 to –21 | 102 (8.4) | 81 (8.5) | 21 (7.7) | 95 (8.3) | 7 (9.0) |
| > –21 to –11 | 429 (35.2) | 349 (36.8) | 80 (29.5) | 411 (36.0) | 18 (23.1) |
| > –11 to –1 | 562 (46.1) | 429 (45.3) | 133 (49.1) | 522 (45.7) | 40 (51.3) |
| > –1 | 109 (8.9) | 77 (8.1) | 32 (11.8) | 98 (8.6) | 11 (14.1) |
| Percentage change from pre-dose mean value |  |  |  |  |  |
| < –40% | 5 (0.4) | 3 (0.3) | 2 (0.7) | 4 (0.4) | 1 (1.3) |
| ≥ –40% but < –30% | 46 (3.8) | 36 (3.8) | 10 (3.7) | 42 (3.7) | 4 (5.1) |
| ≥ –30% but < –20% | 253 (20.8) | 202 (21.3) | 51 (18.8) | 242 (21.2) | 11 (14.1) |
| ≥ –20% but < –10% | 503 (41.3) | 399 (42.1) | 104 (38.4) | 473 (41.5) | 30 (38.5) |
| ≥ –10% but < 0% | 320 (26.3) | 247 (26.1) | 73 (26.9) | 299 (26.2) | 21 (26.9) |
| ≥ 0% | 92 (7.5) | 61 (6.4) | 31 (11.4) | 81 (7.1) | 11 (14.1) |

*bpm* beats per minute, *BBs* beta blockers, *CCBs* calcium channel blockers, *PCCs* pre-existing cardiac conditions or baseline cardiac findings

**Online Resource 3**

Of 2,404 patients assessed by dilated ophthalmoscopy, 19 patients (0.8%) were diagnosed with macular edema in at least one eye during the study or at study completion. Diagnosis was confirmed by a local ophthalmologist in 16 patients (0.7%), of whom 13 (0.5%) discontinued the study drug, while 3 (0.1%) were diagnosed at study completion. Of the 26 patients with MS and diabetes mellitus, 1 (3.8%) was diagnosed with macular edema. This patient had diabetic retinopathy, a risk factor for macular edema, at study entry.

Liver transaminase elevation was reported in 115 patients (4.8%); 14 patients (0.6%) discontinued fingolimod owing to abnormal liver function test results or elevated liver enzymes. Increases of alanine aminotransferase levels threefold and fivefold above the upper limit of normal were reported in 68 (2.8%) and 11 (0.5%) patients, respectively.

There were 117 patients (4.8%) who reported herpes infections, all of which were localized in nature and were mostly (3.1%) attributed to oral herpes. Herpes zoster was reported in 13 patients (0.5%). There were 6 MS relapses reported as SAEs, none of which resulted in study drug discontinuation.

In total, 10 malignancies (0.4%) were reported during the study. These included 7 skin events; not all were malignant (4 patients with basal cell carcinoma, 2 patients with squamous cell carcinoma and 1 patient with Bowen’s disease). In addition there were 3 cases of solid tumor (one patient each): breast cancer, colon cancer and lung neoplasm.
